# Cost-Effectiveness Analysis of Hepatic Arterial Infusion Chemotherapy of Infusional Fluorouracil, Leucovorin, and Oxaliplatin Versus Transarterial Chemoembolization in Patients With Large Unresectable Hepatocellular Carcinoma

**DOI:** 10.3389/fphar.2022.849189

**Published:** 2022-04-26

**Authors:** Haixia Zhang, Xiaohui Zeng, Ye Peng, Chongqing Tan, Xiaomin Wan

**Affiliations:** ^1^ Department of Pharmacy, The Hunan Children’s Hospital, Changsha, China; ^2^ PET-CT Center, The Second Xiangya Hospital of Central South University, Changsha, China; ^3^ Department of Pharmacy, The Second Xiangya Hospital of Central South University, Changsha, China

**Keywords:** FOLFOX-HAIC, TACE, cost-effectiveness, hepatocellular carcinoma, Markov model

## Abstract

**Purpose:** The purpose of this study was to evaluate a cost-effectiveness analysis of hepatic arterial infusion chemotherapy with infusional fluorouracil, leucovorin, and oxaliplatin (FOLFOX-HAIC) as the first-line treatment for patients with large unresectable hepatocellular carcinoma (HCC) compared with transarterial chemoembolization (TACE).

**Methods:** A Markov model was constructed to simulate the first-line treatment, disease recurrence, and survival of patients with large unresectable HCC. Transition probabilities were based on clinical trial data. The costs and health utilities were derived from the public literature. The outputs were total cost, quality-adjusted life year (QALY), and incremental cost-effectiveness ratios (ICER). One-way and probabilistic sensitivity analyses were performed to examine model uncertainty. We also performed subgroup analyses.

**Results:** The results of the base case analysis found that FOLFOX-HAIC increased overall costs by $9,381 and improved effectiveness by 1.01 QALYs compared with TACE. The one-way sensitivity analysis showed that the hazard ratio of progression-free survival and overall survival for FOLFOX-HAIC relative to TACE had the greatest impact on the ICER. Probabilistic sensitivity analysis found that the probability of FOLFOX-HAIC treatment being cost-effective was 99.54% at the willingness-to-pay threshold of $30,552/QALY. Patients in most subgroups favored FOLFOX-HAIC treatment because it had a more than 50% probability of being cost-effective than TACE, except for patients with negative hepatitis B infection.

**Conclusion:** In conclusion, our study found that the FOLFOX-HAIC was a cost-effective option compared to TACE for patients with large unresectable HCC in China.

## Introduction

Primary liver cancer makes up for approximately 4.7% of all cancers worldwide, with approximately 906,000 new cases in 2020 (Sung et al., 2021). Hepatocellular carcinoma (HCC) cases account for approximately 75–85% of primary liver cancer cases (Sung et al., 2021). About 80% of HCC patients worldwide are found in developing countries, and more than half of them are diagnosed in China. (Li et al., 2022). Patients with intermediate-stage of HCC are a heterogenous population, and tumor burdens vary greatly among patients. Currently, the standard treatment of intermediate-stage HCC is transarterial chemoembolization (TACE) (Xie et al., 20192020). However, the efficacy of TACE is related to tumor size, and its complete tumor response rate in large HCC is significantly lower than that in small HCC (25% v 64%) (Golfieri et al., 2013).

The benefit of hepatic arterial infusion chemotherapy (HAIC) with infusional fluorouracil, leucovorin, and oxaliplatin (FOLFOX) in the treatment of large unresectable HCC without vascular invasion or extrahepatic metastasis was recently reported in a randomized phase III study by Qijiong Li et al., which compared this strategy with TACE (Li et al., 2022). The study found that the use of FOLFOX-HAIC significantly improved overall survival (OS) compared with TACE among patients with unresectable large HCC.

The study by Qijiong Li et al. found that FOLFOX-HAIC can improve survival, although this strategy may increase treatment costs. This naturally raises the question of whether FOLFOX-HAIC is a cost-effective strategy. This issue is particularly important because the incidence and mortality of HCC in developing countries are high, and health care decisions in these countries are often made with limited resources. The purpose of this study was to evaluate the cost-effectiveness of FOLFOX-HAIC compared with TACE for the first-line treatment of large unresectable HCC.

## Materials and Methods

### Decision Model

**FIGURE 1:**
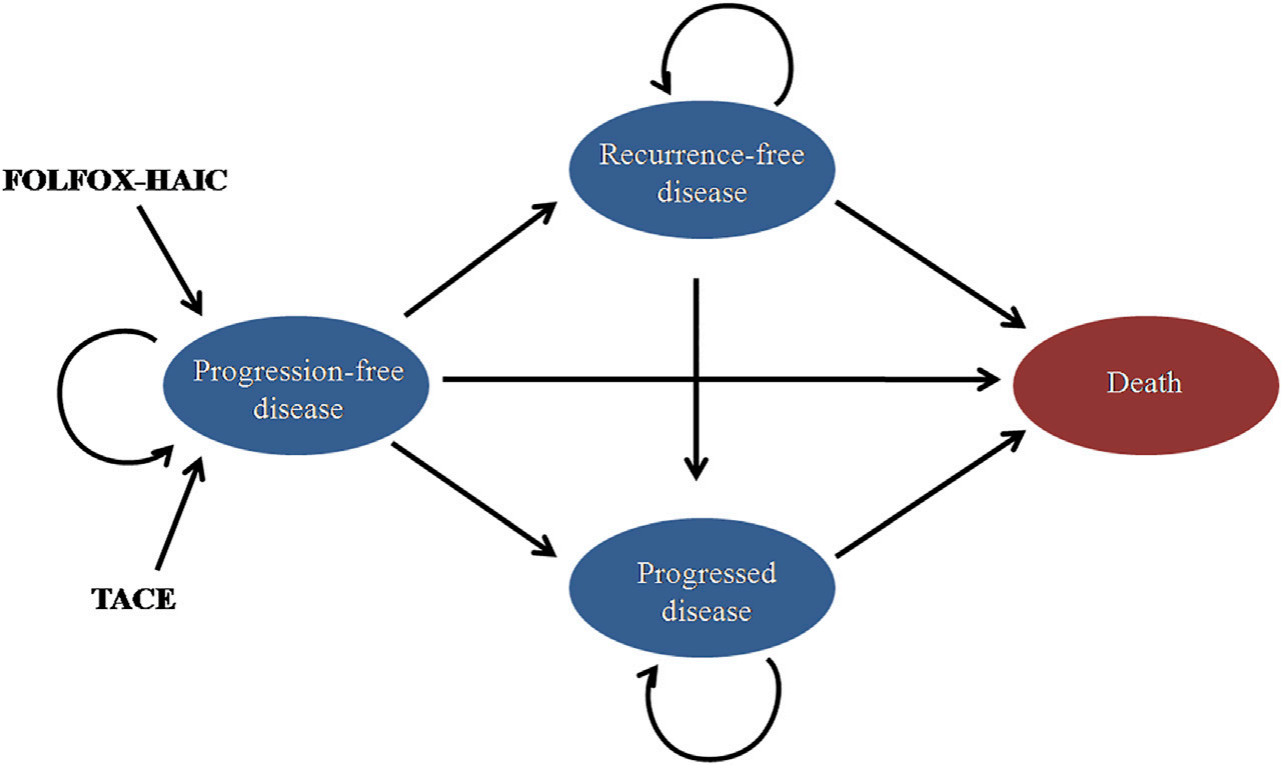
| State-transition diagram for large unresectable hepatocellular carcinoma. The four main health states are represented by ovals. Patients may transition from “progression-free disease” to “recurrence-free disease, ”“progressed disease,” or “death.” TACE, transarterial chemoembolization; FOLFOX-HAIC, hepatic arterial infusion chemotherapy with infusional fluorouracil, leucovorin, and oxaliplatin.

This study compared the cost-effectiveness of FOLFOX-HAIC and TACE for large unresectable HCC. A Markov model was built to simulate the treatment, toxicity, and survival of the patient population. The state-transition diagram shows how the patient’s disease state flows through the model, including progression-free disease (PFD), recurrence-free disease (RFD), progressed disease (PD), and death states (Figure 1). The Markov model was based on the Chinese perspective of the healthcare system and runs on a lifetime horizon by using a 21-day cycle length. The model was constructed using TreeAge Pro 2021 (TreeAge Software, Williamstown, MA) and R.

### Model Probabilities

All patients entering the model were assumed to have large unresectable HCC and received either FOLFOX-HAIC or TACE. The progression-free survival (PFS) and OS data of patients were based on the outcomes of the study by Qijiong Li et al. (Li et al., 2022), and the data outside the study time horizon were obtained by extrapolation using the method described by Guyot et al. (Table 1) (Guyot et al., 2012). The subsequent treatment after progress was modeled based on the outcomes from the method by Qijiong Li et al. (Li et al., 2022). After first-line treatment, 24% of patients without progression in the FOLFOX-HAIC group and 12% in the TACE group underwent hepatectomy. After completing hepatectomy, these patients entered the RFD state. Since the risks of recurrence after hepatectomy in the Qijiong Li et al. study was not available, we assumed a 5-year recurrence rate of approximately 19% in our model based on a study on the prediction of HCC recurrence (Zheng et al., 2017). The 3- and 7-year recurrence rates (15 and 20%) were used as the lower and upper limits for sensitivity analysis, respectively (Zheng et al., 2017). Once cancer recurred after hepatectomy, these patients entered the PD health state. After tumor progression but without stage progression in patients who had not received surgical treatment, treatment crossover was allowed. Other patients with tumor progression were assumed to receive the best supportive care (BSC) until death. In addition, the background mortality rate was also included in the model (Table 2) (National Comprehensive Ca, 2021).

**TABLE 1:** Parameters for the cost-effectiveness model.

Variable	Baseline value	Minimum	Maximum	Reference	Distribution
Log-logistic OS survival model with TACE	Theta = 0.00272126, Kappa = 2.184792	—	—	Li et al. (2022)	—
Lognormal PFS survival model with TACE	Mu = 1.682471, Sigma = 1.119812	—	—	Li et al. (2022)	—
HR of FOLFOX-HAIC group versus TACE group for OS	0.58	0.45	0.75	Li et al. (2022)	Lognormal
HR of FOLFOX-HAIC group versus TACE group for PFS	0.57	0.45	0.72	Li et al. (2022)	Lognormal
Proportion of receiving hepatectomy after FOLFOX-HAIC	0.24	0.19	0.29	Li et al. (2022)	Beta
Proportion of receiving hepatectomy after TACE	0.12	0.10	0.14	Li et al. (2022)	Beta
Proportion of recurrence of HCC	0.19	0.15	0.20	Zheng et al. (2017)	Beta
Proportion of receiving subsequent treatment on BSC after FOLFOX-HAIC	0.95	0.76	1.14	Li et al. (2022)	Beta
Proportion of receiving subsequent treatment on BSC after TACE	0.87	0.70	1.04	Li et al. (2022)	Beta
Proportion of receiving subsequent treatment on TACE after FOLFOX-HAIC	0.05	0.04	0.06	Li et al. (2022)	Beta
Proportion of receiving subsequent treatment on HAIC after TACE	0.13	0.10	0.16	Li et al. (2022)	Beta
FOLFOX-HAIC arm: Incidence of Grade ≥3 AEs					
Elevated ALT	0.08	0.06	0.10	Li et al. (2022)	Beta
Elevated AST	0.18	0.14	0.22	Li et al. (2022)	Beta
Vomiting	0.06	0.05	0.07	Li et al. (2022)	Beta
TACE arm: Incidence of Grade ≥3 AEs					
Elevated ALT	0.19	0.15	0.23	Li et al. (2022)	Beta
Elevated AST	0.29	0.23	0.35	Li et al. (2022)	Beta
Vomiting	0.05	0.04	0.06	Li et al. (2022)	Beta
Utility					
PFD	0.76	0.61	0.91	Li et al. (2021)	Beta
PD	0.68	0.54	0.82	Li et al. (2021)	Beta
AE disutility					
Elevated ALT/AST	0	0	0	Li et al. (2021)	Beta
Vomiting	0.05	0.04	0.06	Li et al. (2021)	Beta
AE cost, $/event					
Elevated ALT/AST	43	35	52	Li et al. (2021)	Gamma
Vomiting	49	39	59	Li et al. (2021)	Gamma
Discount rate	0.03	0.01	0.05	Sanders et al. (2016)	Uniform
Drug cost, US $/cycle					
Oxaliplatin	426	340	511	Li et al. (2021)	Gamma
Fluorouracil	524	419	629	Li et al. (2021)	Gamma
Leucovorin	24	19	29	Li et al. (2021)	Gamma
HAIC	1,850	1,480	2,220	Li et al. (2021)	Gamma
TACE	1,929	1,543	2,315	Chen et al. (2018)	Gamma
Hepatectomy	9,022	7,218	10,827	Li et al. (2021)	Gamma
Hospitalization	384	307	460	Li et al. (2021)	Gamma
BSC	363	291	436	Li et al. (2021)	Gamma

OS, overall survival; PFS, progression-free survival; HR, hazard ratio; TACE, transarterial chemoembolization; FOLFOX-HAIC, hepatic arterial infusion chemotherapy with infusional fluorouracil, leucovorin, and oxaliplatin; AEs, adverse events; PFD, progression-free disease; PD, progressed disease; BSC, best supportive car.

**TABLE 2:** Background mortality rate.

Age	Background mortality rate	Age	Background mortality rate
<1 year	0.006763658	45–49 years	0.012327216
1–4 years	0.001144414	50–54 years	0.01956602
5–9 years	0.000924507	55–59 years	0.030500498
10–14 years	0.000969999	60–64 years	0.048778564
15–19 years	0.00179698	65–69 years	0.078138957
20–24 years	0.002810805	70–74 years	0.137236768
25–29 years	0.003052746	75–79 years	0.221445846
30–34 years	0.004239707	80–84 years	0.371374039
35–39 years	0.006187994	85+ years	1
40–44 years	0.009315495	—	—

### Costs

This analysis was conducted from the Chinese perspective of the healthcare system. We included directed medical costs, including the cost of first-line and subsequent treatments, hospitalization, hepatectomy, and management of grade 3–4 adverse events (Table 1). Based on the study of Qijiong Li et al. (Li et al., 2022), the mean number of TACE treatments for each patient was 2, once every six weeks. The cost of TACE was derived from a previously published study (Chen et al., 2018). For the FOLFOX-HAIC regimen, the microcatheter was first advanced into the patient’s hepatic artery, and then the drugs were infused through the hepatic artery: oxaliplatin, 130 mg/m^2^; leucovorin, 400 mg/m^2^; and fluorouracil, 400 mg/m^2^ bolus and 2,400 mg/m^2^ over 24 h. Based on the study of Qijiong Li et al. (Li et al., 2022), the mean number of HAIC treatments for each patient was 3.6, once every three weeks. The drug prices and hepatic artery catheterization fees were derived from published literature (Li et al., 2021). We also considered the cost of treatment-related grade 3–4 adverse events with a higher incidence of more than 5%, including elevated ALT/AST and vomiting (Li et al., 2021). The total cost of AEs was the sum of the incidence of each AE multiplied by its associated unit cost. All costs were adjusted to USD 2020 according to the Consumer Price Index (Powles et al., 2020).

### Outcome Measures

The outcomes were measured in quality-adjusted life years (QALYs), which were calculated by multiplying the life years (LYs) and health utility. The health utility reflected the patient’s quality of life, ranging from 0 (death) to 1 (perfect health). According to published literature in the Chinese setting, the utility value in the state of PFD and RFD after hepatectomy was assumed to be 0.76, and the utility value in the state of PD was 0.68 (Qin et al., 2018; Li et al., 2021). In addition, the reduction in utility values caused by grade 3–4 adverse events with a higher incidence of more than 5% was also considered in the model. We also performed a sensitivity analysis on the uncertainty of the utility value. An annual discount rate of 3% was used for all health utilities and costs (Sanders et al., 2016).

### Analysis

Our results were measured by the incremental cost-effectiveness ratio (ICER), which was the incremental cost of each additional QALY between the two treatments. If the ICER fell under the willingness-to-pay (WTP) threshold, the treatment was considered to be cost-effective. According to the recommendations of the World Health Organization, we have adopted three times the per-capita gross domestic product (GDP) of China in 2020 ($30,552/QALY) as the WTP threshold (Powles et al., 2020; Murray et al., 2000). In addition, we conducted one-way sensitivity analyses for each variable to determine the factors that directly influenced the ICER. The range of all parameters was their 95% CIs derived from the literature or assumed to be ±20% of the baseline value. Finally, we also conducted a probabilistic sensitivity analysis through the Monte Carlo simulation with 10,000 iterations. The cost parameter estimation was modeled with gamma distribution. All probability and health utility parameters were modeled with Beta distribution. The hazard ratio parameter was modeled with a lognormal distribution. Base-case values of each variable and their upper and lower limits and distribution are shown in Table 1. We also performed subgroup analyses of all patients by using subgroup-specific hazard ratios (HRs) reported in the study of Qijiong Li et al. based on the method of Hoyle et al. (Hoyle et al., 2010).

## Results

### Base Case Analysis

The results of the base case analysis found that the total cost of the FOLFOX-HAIC group was $19,788 compared with $10,407 for the TACE group. When considering the outcome, the FOLFOX-HAIC strategy yielded 2.28 QALYs compared with 1.27 QALYs for TACE, although FOLFOX-HAIC increased $9,381 but added 1.01 QALYs compared with TACE, resulting in an ICER of $9,247/QALY.

### Sensitivity Analysis

**FIGURE 2:**
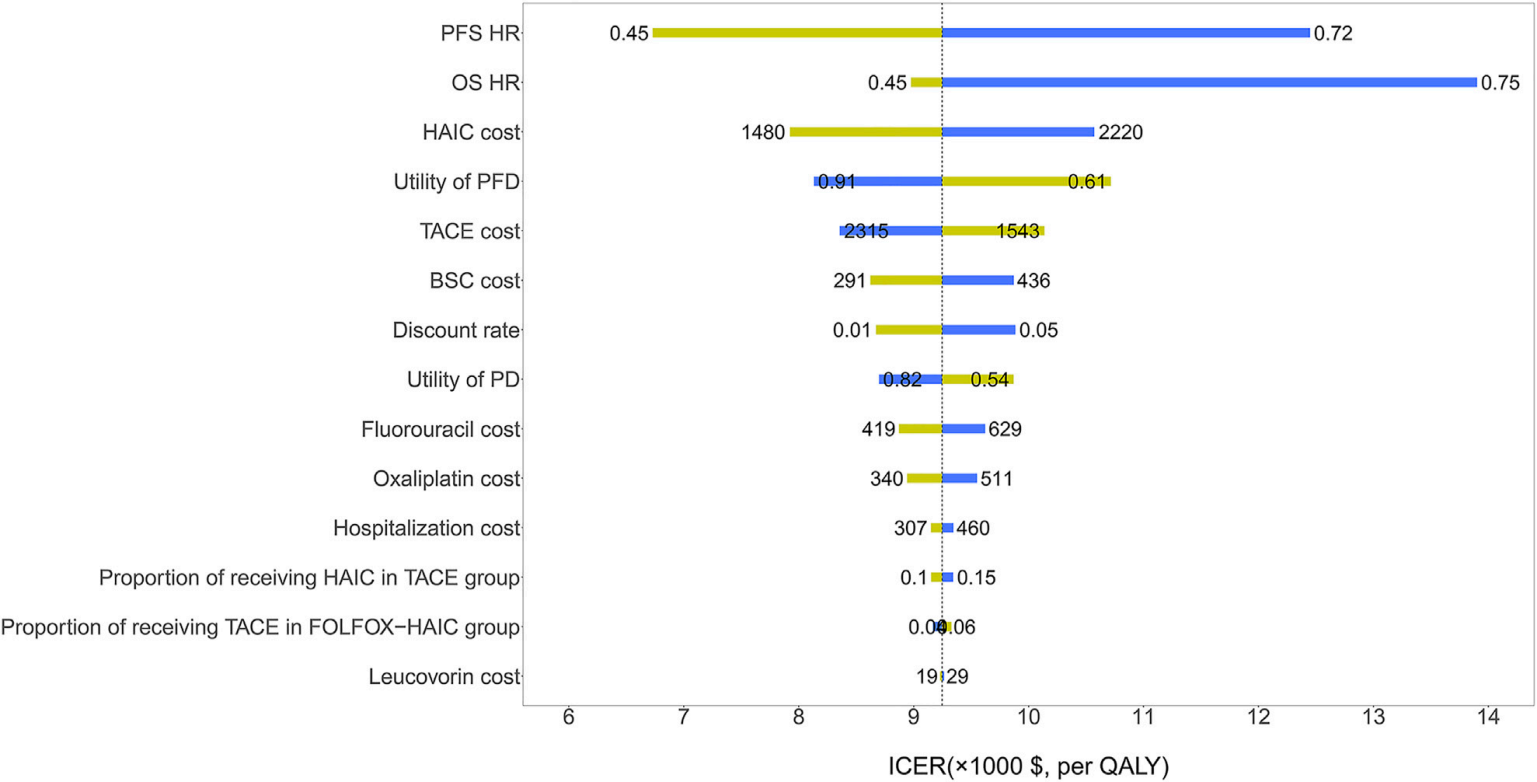
| One-way sensitivity analysis results of the FOLFOX-HAIC strategy versus TACE strategy in all patients with large unresectable hepatocellular carcinoma. The tornado diagram shows the impact of varying model parameters on the incremental cost-effectiveness ratio of the FOLFOX-HAIC strategy compared to the TACE strategy. The dotted line intersecting the blue and yellow bar represents the ICER of $30,552/QALY from the base case results. OS, overall survival; PFS, progression-free survival; HR, hazard ratio; TACE, transarterial chemoembolization; FOLFOX-HAIC, hepatic arterial infusion chemotherapy with infusional fluorouracil, leucovorin, and oxaliplatin; PFD, progression-free disease; PD, progressed disease; BSC, best supportive care.

**FIGURE 3:**
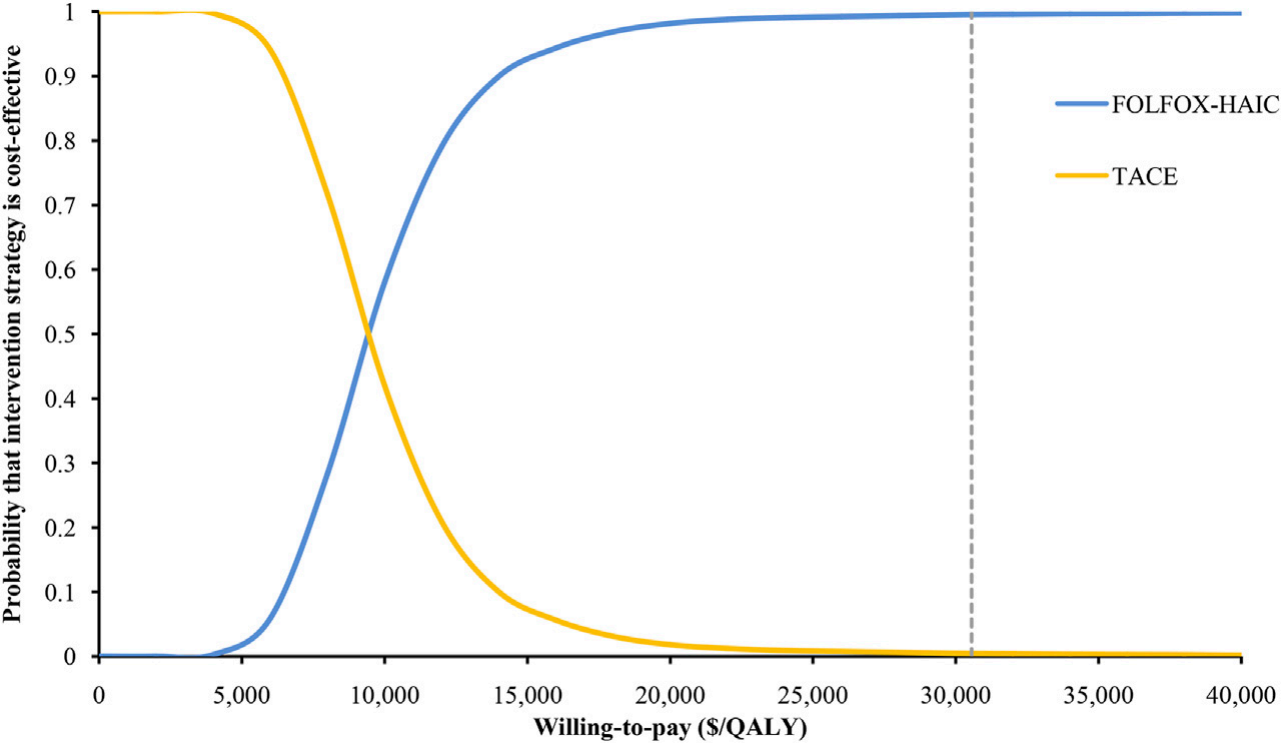
| Cost-effectiveness acceptability curves for the FOLFOX-HAIC strategy compared to the TACE strategy. This plot represents the results of a probabilistic sensitivity analysis showing the probability of cost-effectiveness of the FOLFOX-HAIC strategy versus the TACE strategy in large unresectable hepatocellular carcinoma. TACE, transarterial chemoembolization; FOLFOX-HAIC, hepatic arterial infusion chemotherapy with infusional fluorouracil, leucovorin, and oxaliplatin; QALY, quality-adjusted life year.

The HR of PFS and OS for FOLFOX-HAIC relative to TACE had the greatest impact on the ICER (Figure 2). Other parameters, such as the cost of HAIC and the utility of PFD, had relatively little impact on the ICER. Of note, all ICERs remained below the WTP threshold of $30,552/QALY. The probabilistic sensitivity analysis results are shown in the cost-effectiveness acceptability curve (Figure 3); the probability that FOLFOX-HAIC is cost-effective compared with TACE was 99.54% at WTP thresholds of $30,552/QALY.

### Subgroup Analysis

For most subgroups, the ICER of FOLFOX-HAIC compared with TACE was less than the WTP threshold of $30,552/QALY, ranging from $5,210/QALY (probabilities of cost-effectiveness, 99.75%) in patients with age ≤50 years to $30,069/QALY (probabilities of cost-effectiveness, 56.19%) in patients with Child–Pugh grade A (6 points) (Table 3). Only in the subgroup of patients with negative hepatitis B infection, the ICER of FOLFOX-HAIC compared with TACE was higher than the WTP threshold of $30,552/QALY, which was $62,762/QALY (probabilities of cost-effectiveness, 48.65%)

**TABLE 3:** Results for subgroup analyses.

Subgroup	PFS HR (95% CI)	OS HR (95% CI)	ICER ($/QALY)	Cost-effectiveness probability
Age				
≤50 years	0.40 (0.27–0.58)	0.46 (0.31–0.69)	5,210	99.75%
>50 years	0.71 (0.52–0.96)	0.67 (0.49–0.94)	14,253	90.36%
Sex				
Male	0.55 (0.42–0.70)	0.60 (0.46–0.78)	9,004	97.61%'
Female	0.85 (0.42–1.72)	0.48 (0.21–1.09)	12,196	92.65%
ECOG PS				
0	0.51 (0.37–0.69)	0.58 (0.41–0.80)	7,924	98.25%
1	0.67 (0.46–0.98)	0.57 (0.38–0.85)	9,174	98.33%
Child–Pugh score				
A (5 points)	0.50 (0.38–0.66)	0.53 (0.40–0.71)	7,390	99.25%
A (6 points)	0.89 (0.52–1.52)	0.81 (0.46–1.41)	30,069	56.19%
Hepatitis B infection				
Positive	0.55 (0.42–0.70)	0.55 (0.44–0.72)	8,599	100.00%
Negative	0.79 (0.39–1.59)	0.91 (0.44–1.88)	62,762	48.65%
AFP, ng/mL				
≤400	0.64 (0.46–0.89)	0.63 (0.44–0.90)	11,546	95.61%
>400	0.48 (0.35–0.68)	0.53 (0.37–0.76)	6,958	99.08%
Tumor size, cm				
≤10	0.61 (0.44–0.84)	0.55 (0.38–0.79)	10,049	98.77%
>10	0.52 (0.37–0.74)	0.60 (0.42–0.86)	8,358	97.56%
Tumor number				
≤3	0.50 (0.35–0.71)	0.52 (0.36–0.77)	7,367	99.47%
>3	0.69 (0.50–0.95)	0.66 (0.47–0.93)	13,464	91.88%

AFP, alpha-fetoprotein; ECOG, Eastern Cooperative Oncology Group; HR, hazard ratio; OS, overall survival; PFS, progression-free survival; ICER, incremental cost-effectiveness ratio; QALY, quality-adjusted life years.

## Discussion

In recent years, the effect of drug treatment for liver cancer has been unsatisfactory. Researchers are constantly exploring new treatment options (Ho et al., 2018). The reports on the clinical benefit of FOLFOX-HAIC in the study of Qijiong Li et al. have aroused the interest of oncologists and patients (Li et al., 2022). However, the cost of liver cancer treatment accounts for a significant share of cancer health expenditure in China, and public health expenditure must be allocated according to the best societal value. Therefore, it is inevitable to evaluate the economics of a new treatment option for liver cancer. Our study performed a cost-effectiveness analysis of FOLFOX-HAIC versus TACE as the first-line treatment for patients with large unresectable HCC. Based on the study of Qijiong Li et al., our analysis showed that FOLFOX-HAIC for the treatment of large unresectable HCC was a cost-effective option at the WTP threshold of $30,552/QALY. Patients in most subgroups favored FOLFOX-HAIC treatment because it had a more than 50% probability of being cost-effective compared to TACE, except for patients with negative hepatitis B infection.

As far as we know, there is still very little literature on the cost-effectiveness evaluation of HAIC in the treatment of liver cancer. Only one Chinese-based study by Meiyue Li et al. evaluated the cost-effectiveness of the hepatic arterial infusion of FOLFOX plus sorafenib (SoraHAIC) in the treatment of advanced hepatocellular carcinoma with portal vein invasion (Li et al., 2021). The results showed that SoraHAIC was not a cost-effective option compared to sorafenib in low- and medium-income areas of China, while the probability of being cost-effective in high-income areas of China (Beijing) was 38.8%. Currently, there are no published studies on the economics of FOLFOX-HAIC versus TACE in the first-line treatment of large unresectable HCC. Therefore, the economic evaluation of determining the optimal treatment plan by comprehensively considering the effectiveness and costs of treating large unresectable HCC patients was of great significance for China, which has a large number of patients and subsequently a heavy medical burden.

The nature of FOLFOX-HAIC that prolongs the survival of unresectable large HCC was the driving force for the model’s prediction. Our one-way sensitivity analysis showed that the HR of PFS and OS had the most influence on the model. This result indicated that FOLFOX-HAIC had a higher probability of being cost-effective for patients with age ≤50 years, alpha-fetoprotein (AFP) >400 ng/ml, tumor number ≤3, Child–Pugh grade A (5 points), ECOG score of 0, tumor size >10 cm, and positive hepatitis B infection. However, the probability of FOLFOX-HAIC being considered cost-effective was less than 50% for the subgroup of patients with negative hepatitis B infection.

The limitations of our study were mainly caused by the quality of the input information of the model. Although the study by Qijiong Li et al. provided a large portion of patient survival data, the long-term PFS and OS survival estimates of patients were fitted with the lognormal and log-logistic functions, respectively. This was an inevitable limitation of this study. The model should be validated when actual long-term survival data could be available in the future. Other model input parameters, such as health utilities and costs, were derived from heterogenous sources, which might affect the results of the model. In addition, due to the lack of reports on the quality of life data in the FOLFOX-HAIC and TACE groups, we used the same health utility value for the two treatment strategies and then adjusted for the reduction in utility value caused by adverse events of grade ≥3. However, sensitivity analysis was performed on these parameters, which suggested that these parameters did not have a significant impact on our results. A final limitation was related to the treatment cycle of FOLFOX-HAIC and TACE. Considering that each patient received a different cycle of treatment, the treatment cycle estimation in our model was based on the mean number of treatments received by patients in the study by Qijiong Li et al., which might be different from the actual situation of patients. Nevertheless, despite these limitations, our study still reflected the current general situation of the treatment for large unresectable HCC patients in China and could provide valuable reference information for Chinese clinicians and policy-makers.

In conclusion, our study found that the FOLFOX-HAIC was a cost-effective option compared to TACE for patients with large unresectable HCC in China.

## Data Availability

The original contributions presented in the study are included in the article/Supplementary Material; further inquiries can be directed to the corresponding authors.
